# Radiation-Induced Synthesis of Polymer Networks Based on Thermoresponsive Ethylene Glycol Propylene Glycol Monomers

**DOI:** 10.3390/gels11070488

**Published:** 2025-06-24

**Authors:** Andjelka Stolic, Zorana Rogic Miladinovic, Maja Krstic, Georgi Stamboliev, Vladimir Petrovic, Edin Suljovrujic

**Affiliations:** 1 Vinca Institute of Nuclear Sciences-National Institute of the Republic of Serbia, University of Belgrade, Mike Petrovica Alasa 12-14, P.O. Box 522, 11001 Belgrade, Serbia; andjelka.jolic@vin.bg.ac.rs (A.S.); zoranar@vin.bg.ac.rs (Z.R.M.); majamicic@vin.bg.ac.rs (M.K.);; 2 Global Supply Line, Adelaide 5109, Australia; 3 Faculty of Science, Department of Chemistry, University of Kragujevac, 34000 Kragujevac, Serbia

**Keywords:** thermoresponsive polymer, hydrogel, radiation synthesis, water/ethanol, EGPG unit

## Abstract

In this paper, different poly((ethylene glycol)-(propylene glycol)) methacrylate (P(EGPG)MA) hydrogels were synthesized by gamma-radiation-induced polymerization and crosslinking from a monomer–bisolvent mixture using the following monomers: (ethylene glycol)_6_ methacrylate (EG_6_MA), ((ethylene glycol)_6_-(propylene glycol)_3_) methacrylate (EG_6_PG_3_MA), ((propylene glycol)_6_-(ethylene glycol)_3_) methacrylate (PG_6_EG_3_MA), and (propylene glycol)_5_ methacrylate (PG_5_MA), along with different water/ethanol compositions as the solvent. The monomer–bisolvent mixture was exposed to various radiation doses (5, 10, 15, 25, and 50 kGy). Considerable emphasis was placed on optimizing and tuning the reaction conditions necessary for the fabrication of methacrylic networks with pendant EGPG terminals. A further investigation was conducted on the effects of monomer composition, different preparation conditions, and radiation processing on thermal properties, microstructure, swelling behavior, and volume phase transition. Special attention was dedicated to PPG_6_EG_3_MA hydrogel, whose volume phase transition temperature is near physiological temperatures. This study identifies an optimal radiation dose and a water/ethanol solvent ratio for the synthesis of the radiation-induced hydrogels. Employing ionizing radiation within the sterilization dose range enables the simultaneous fabrication and sterilization of these hydrogels, offering an efficient production process. The findings provide new insights into the role of bisolvent composition on hydrogel formation and properties, and they present practical guidelines for optimizing hydrogel synthesis across a wide range of applications.

## 1. Introduction

Stimuli-responsive hydrogels, which undergo structural or mechanical changes in response to environmental cues such as light, temperature, or pH, have become pivotal in various applications, including drug delivery, sensing, and regenerative medicine. These hydrogels offer dynamic functionality that can be tailored by adjusting their response. As an exogenous stimulus, temperature enables remote non-invasive control over temperature-sensitive hydrogels [1]. The temperature-dependent phase separation of monomer/polymer in aqueous solutions, changes in solubility, and the transition from a solvated coil to a collapsed globule [2,3] allow these materials to exhibit a lower critical solution temperature (LCST) or volume phase transition temperature (VPTT) when polymerized and crosslinked from their soluble state. These phase transitions depend on the hydrophilic/hydrophobic balance of the polymer, influencing swelling behavior and responsiveness.

The primary subject of this study is thermoresponsive hydrogels with pendant ethylene glycol (EG) and propylene glycol (PG) terminals. Both EG and PG impart thermoresponsive properties, making them ideal for modulating the physical characteristics of hydrogels. These terminals have a significant influence on the swelling behavior, phase transitions, and overall stability of hydrogels, particularly under varying environmental conditions.

Poly(ethylene glycol) (PEG) is widely used in tissue engineering scaffolds due to its non-immunogenic, non-cytotoxic, and bio-inert properties [4,5,6,7]. Its ability to prevent protein adsorption and cell adhesion has made PEG-based hydrogels a popular choice for drug delivery applications [8,9]. PEG’s hydrophilicity, high chain mobility, and steric stabilization effects further enhance its role in preventing protein adsorption and platelet adhesion [10]. Building on PEG’s advantageous properties, alternative thermoresponsive polymers, such as acrylate- or methacrylate-based materials with short-to-intermediate oligo(ethylene glycol) (OEG) side chains, have emerged as promising PEG analogs with lower LCST values [11].

Poly(propylene glycol) (PPG) is a thermoresponsive polymer with an LCST that is influenced by its molecular weight [12]. Moreover, PG-based monomers exhibit lower LCSTs than their EG-based counterparts due to increased hydrophobicity from an additional methyl group, which reduces hydrogen bonding with water and limits aqueous solubility. As previously reported [13], increasing OPGMA concentration further decreases LCST and leads to persistent turbidity above 3 wt%, indicating limited solvation and aggregation driven by favored, but incomplete, interactions with water. Although PGs have attracted less attention due to their hydrophobicity, their analogs, such as oligo(propylene glycol) methacrylate (OPGMA), and block copolymers (e.g., Pluronic and Poloxamer) have shown utility in industrial applications [14,15].

Our previous study revealed that OPGMA forms a thermoresponsive hydrogel with a VPTT of between 13 and 16 °C [16]. However, despite its good biocompatibility, low water solubility, and a VPTT below the room temperature limits its biomedical use. Efforts to shift the hydrogel’s VPTT toward physiological temperatures include copolymerization with monomers like 2-hydroxyethyl methacrylate (HEMA), itaconic acid (IA), or oligo(ethylene glycol) methacrylate (OEGMA) [13,17].

Oligo(alkylene glycols) with varying numbers of EG and PG units, such as EG_X_PG_Y_MA [18,19], are used to modulate the LCST and, consequently, the VPTT of hydrogels. The methacrylate (MA) group facilitates efficient polymerization, while the EG_X_PG_Y_ terminals provide thermoresponsive properties [20]. Instead of combining hydrophilic and hydrophobic monomers in a copolymer, a single monomer with different numbers of EG and PG units offers targeted control over the amphiphilic balance within the monomer structure. In contrast to OEGMA, the amphiphilic alternating monomer EG_X_PG_Y_MA exhibits lower solubility in water, necessitating a distinct and more complex approach for synthesis.

Using a water/alcohol mixture as a solvent instead of pure water significantly enhances the solubility of amphiphilic alternating monomers and polymers [17,21,22]. Ethanol, in particular, demonstrates unusual packing characteristics in water, as reported by Parke et al. [23]. While extensive literature exists on the structural features of water/ethanol solutions and the nature of its intermolecular bonds [24,25,26], the conclusions are often controversial [26,27]. Interestingly, the addition of some monomers and polymers exhibits solubility in a water/ethanol mixture, despite being insoluble in either solvent individually at room temperature [28,29]. This co-solvency effect is likely due to changes in the local conformation of ethanol and water molecules around the monomer/polymer unit [22]. The system becomes more intricate when preparing hydrogels, as physically and/or chemically crosslinked networks are typically formed from more concentrated monomer/polymer solutions (often 5–10 wt% or higher) [3,30]. Generally, adding water or ethanol to pre-polymerized mixtures results in less dense hydrogel networks than without a solvent [30,31]. At equivalent weight ratios, water disrupts the final crosslinking of PEG-based hydrogels more than ethanol, although it accelerates the free-radical polymerization rate during the early stages of crosslinking to a greater extent than ethanol [31]. PEG in water/ethanol mixtures has also been investigated by Ravi et al. [32], who observed a prolonged induction phase in 100% ethanol, attributed primarily to the increased solubility of oxygen, a free-radical scavenger. While the mechanisms by which water/ethanol and other co-solvents influence polymerization are not yet fully clarified, their use in the synthesis of complex multi-component hydrogels is evident [33,34].

Building on strategies for synthesizing hydrogels using co-solvents, gamma radiation was employed as the primary method for sample synthesis in this study. This ionizing radiation technique efficiently crosslinks polymers without toxic chemical initiators or catalysts, resulting in cleaner and more biocompatible hydrogels [35]. The high-energy gamma rays penetrate deeply into materials, enabling uniform crosslinking and offering better control over the hydrogel’s final properties [36]. Additionally, gamma radiation can optimize mechanical properties, such as compressive strength and swelling ratio, by tuning the radiation dose and polymer concentration [37]. It also facilitates simultaneous sterilization, which is particularly beneficial for biomedical applications [35]. The versatility of gamma radiation extends to both natural and synthetic polymers, enabling the creation of hydrogels with tailored properties for specific applications [38]. While electron beam irradiation can also be used, gamma radiation is more effective for thicker or denser hydrogel formulations. Overall, gamma radiation offers a clean, efficient, and controllable method for producing tailored hydrogels for various biomedical applications [35,37,38].

In this study, we aimed to optimize the synthesis of poly((propylene glycol)_6_-(ethylene glycol)_3_) methacrylate (PPG_6_EG_3_MA) and poly((ethylene glycol)_6_-(propylene glycol)_3_) methacrylate (PEG_6_PG_3_MA) thermoresponsive networks with pendant block ethylene glycol propylene glycol (EGPG) terminals. Various synthesis conditions were explored to achieve this optimization. To establish a baseline for comparison, pure EG and PG methacrylate monomers were used, and corresponding methacrylate hydrogels with EG and PG pendant chains, poly(ethylene glycol)_6_ methacrylate (PEG_6_MA), and poly(propylene glycol)_5_ methacrylate (PPG_5_MA) were synthesized.

Our primary objective was to identify the optimal solvent system and gamma radiation dose for hydrogel preparation. To achieve this, we dissolved the monomers in different water-to-ethanol ratios, systematically varying the solvent composition to determine the most effective mixture for synthesis. Following this, we exposed the most promising monomer/solvent solution to varying gamma radiation doses to assess their impact on gel content. The goal was to identify the radiation dose that would yield the highest gel content, leading to the development of hydrogels with desired properties for a wide range of applications.

UV-Vis spectroscopy was employed to monitor the cloud point (CP) as a function of solvent composition and temperature to assess the solubility behavior of the monomer with varying temperature and water content in the bisolvent system, and to determine the monomer’s lower critical solution temperature (LCST).

After synthesis, we characterized all samples, monomers, hydrogels, and xerogels using Fourier Transform Infrared (FTIR) spectroscopy. To evaluate the thermal properties and thermosensitivity of the synthesized hydrogels, we performed Differential Scanning Calorimetry (DSC) analysis and conducted a swelling test, which provided insight into the hydrogel’s response to temperature changes. Finally, we used Scanning Electron Microscopy (SEM) to examine the morphology and structure of the hydrogels, confirming their thermoresponsive behavior.

## 2. Results and Discussion

**Figure 1:**
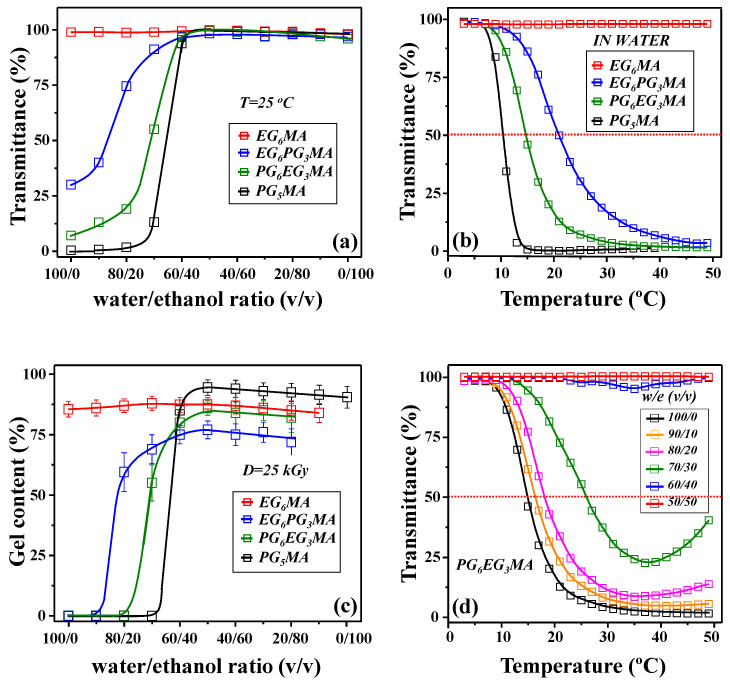
(**a**) UV-Vis transmittance profile for 10 wt% of EG_6_MA, EG_6_PG_3_MA, PG_6_EG_3_MA, and PG_5_MA monomers at 650 nm against different water/ethanol ratios recorded at room temperature. (**b**) Transmittance profile at 650 nm for 10 wt% of EG_6_MA, EG_6_PG_3_MA, PG_6_EG_3_MA, and PG_5_MA monomers in water against temperature. (**c**) Gel content for an absorbed dose of 25 kGy for PEG_6_MA, PEG_6_PG_3_MA, PPG_6_EG_3_MA, and PPG_5_MA hydrogels. (**d**) Transmittance profile at 650 nm for 10 wt% PG_6_EG_3_MA monomers in different water/ethanol ratios. The red horizontal dotted lines highlight the theoretically determined LCST values from the cloud point method.

The optical transmittance, LCST, and phase separation behavior of each monomer in solutions with varying water/ethanol ratios were examined through UV-Vis measurements. The transmittance of the monomer (EG_6_MA, EG_6_PG_3_MA, PG_6_EG_3_MA, and PG_5_MA) solutions as a function of the water/ethanol ratio (from pure water, increasing by 10 volume percent of the ethanol until a pure ethanol solvent was obtained) at room temperature is presented in Figure 1a. The observed trends suggest that, for the EG_6_MA monomer, the transmittance in pure water is nearly 100% and remains unchanged with an increase in ethanol content. In contrast, for EG_6_PG_3_MA, PG_6_EG_3_MA, and PG_5_MA, the transmittance increases as the ethanol content rises. This behavior can be attributed to the underlying interactions in a water/ethanol solution. Specifically, the formation of cage-like structures around the hydrophobic alkyl end of the ethanol molecule influences these interactions, while the hydrophilic hydroxyl group participates in hydrogen bonding with water molecules [23]. At lower ethanol concentrations, stronger water/water hydrogen bonds are formed, stabilizing the cage-like structure of bulk water. As the ethanol content increases, the available interstitial spaces become saturated, causing ethanol–water interactions to be replaced by ethanol–ethanol hydrophobic interactions. This results in the formation of ethanol chains or rings, which are reflected in a rapid increase in the apparent specific volume [23].

Thermoresponsiveness and miscibility temperature were determined using the CP method [39]. The mechanisms of phase separation of polymeric aqueous solutions, in the case of LCST, are driven by entropy, with phase separation at certain temperatures being entropically favorable, mainly due to hydrogen-bonding interactions. An increase in temperature results in the breaking of hydrogen bonds, polymer chains fold, and phase separation occurs as the gain in entropy becomes more favorable compared to enthalpy.

Figure 1b presents the transmittance profiles for 10 wt% of EG_6_MA, EG_6_PG_3_MA, PG_6_EG_3_MA, and PG_5_MA monomers dissolved in water as a solution, measured over the temperature range from 3 to 50 °C. These profiles provide insight into the thermoresponsive behavior of the monomers, which is crucial for understanding the preparation of physically and chemically crosslinked hydrogels commonly used in biomedical applications.

In general, hydrogels for biomedical applications are prepared using more concentrated monomer solutions, typically no less than 5 wt%, with many formulations reaching up to 10 wt% of monomer [3,30]. Therefore, this study focuses on evaluating only monomer–bisolvent mixtures containing 10 wt% of monomers, as it reflects the typical concentration range used in such applications. The transmittance profile of EG_6_MA remains almost constant at 100% across the measured temperature range, which is attributed to its complete dissolution in water. As for the PG_5_MA, the transmittance profile decreases rapidly to nearly zero with increasing temperature, while the profiles of PG_6_EG_3_MA and EG_6_PG_3_MA lie between these extremes. PG_5_MA, PG_6_EG_3_MA, and EG_6_PG_3_MA monomers exhibit similar transmittance profiles typical for thermoresponsive monomers/polymers and have an LCST of below room temperature (10.4, 14.8, and 21.1 °C, respectively, as shown in Table 1). This suggests that increasing the EG content in EGPG terminals significantly raises the LCST, although EG_6_PG_3_MA, despite having a higher EG content, shows low solubility in water at room temperature.

Measuring the gel content provides a reliable method for evaluating the extent of crosslinking in polymeric hydrogels, allowing for the determination of the proportion of crosslinked polymer chains within the hydrogels [40]. As shown in Figure 1c, by changing solvent composition, the gel content remains nearly constant at around 88% for PEG_6_MA. In contrast, the other samples exhibit a sharp increase in gel content with an increase in ethanol content in the water/ethanol solution. This abrupt rise shifts toward a higher ethanol share as the hydrophobicity of the pendant chain increases. Specifically, a gel content of around 60% was reached for PEG_6_PG_3_MA, at an 80/20 water-to-ethanol ratio, and for PPG_6_EG_3_MA at 70/30, while gel content of around 85% PPG_5_MA was reached at 60/40. To achieve saturation in gel content, the ethanol concentration in the water/ethanol (*v*/*v*) solution must exceed 40%. The highest gel content values, regardless of the monomer type, were obtained with an equal volumetric water/ethanol ratio. However, further increases in ethanol content led to a gradual and consistent decrease in gel content, accompanied by a significant rise in the fragility of hydrogels [32,41,42]. This behavior aligns with findings reported in the literature, where similar trends were observed in the mechanical properties of PEG(M)A hydrogels when varying solvent compositions during crosslinking. Specifically, our previous study suggests that ethanol alters the polymerization and crosslinking rates differently, resulting in the formation of distinct network structures [17].

Considering these findings, along with the limitations discussed, it can be concluded that the use of a 50/50 (*v*/*v*) water/ethanol solvent ratio is the most effective approach for producing EG_X_PG_Y_MA- and OPGMA-based hydrogels, offering optimal gel content (above 75%) and mechanical properties.

For all investigated monomers, an increase in ethanol content in the solution (Figure 1a) leads to a rise in transmittance. This increase shifts towards higher temperatures (resulting in a later onset) and becomes less steep, as shown for the PG_6_EG_3_MA monomer in Figure 1d. The transmittance for a 60/40 (*v*/*v*) water/ethanol solution remains above 95% at room temperature and throughout the entire measured temperature range, confirming its good solubility. Overall, the results confirm that adding alcohol is essential for polymer/hydrogel synthesis from thermoresponsive monomers with an LCST below the synthesis temperature. Moreover, this step improved monomer solubility and enhanced polymerization and crosslinking efficiency during irradiation [16,43]. This increase is attributed to the effect of alcohol and the rise in its concentration, which alters the phase behavior of the system. Specifically, the system exhibits behavior characteristics of the upper critical solution temperature (UCST), leading to improved miscibility and higher transmittance with increasing temperature [44].

**Figure 2:**
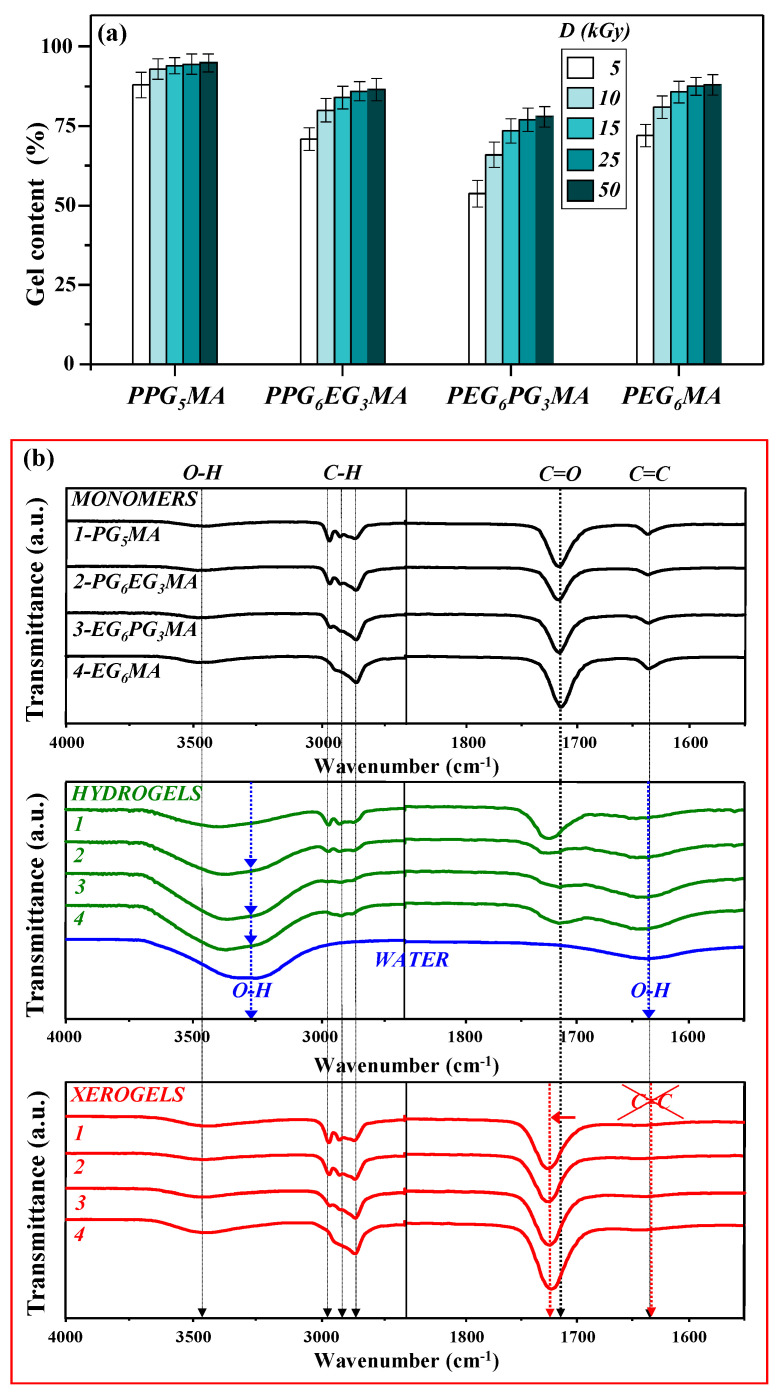
(**a**) Gel content of PPG_5_MA, PPG_6_EG_3_MA, PEG_6_PG_3_MA, and PEG_6_MA hydrogels as a function of applied absorbed dose; (**b**) FTIR spectra of monomers/hydrogels/xerogels in the single bond region (3500–2500 cm^−1^) and double bond region (2000–1500 cm^−1^). The scans were shifted vertically for clarity.

Based on our findings, we employed a 50/50 (*v*/*v*) water/ethanol solution to explore the effect of absorbed dose on the gel content of PPG_5_MA, PPG_6_EG_3_MA, PEG_6_PG_3_MA, and PEG_6_MA hydrogels. A series of monomer–bisolvent mixtures (PG_5_MA, PG_6_EG_3_MA, EG_6_PG_3_MA, and EG_6_MA) with 10 wt% monomers in a 50/50 (*v*/*v*) water/ethanol solution were exposed to five different absorbed doses of gamma radiation (5, 10, 15, 25, and 50 kGy). The effect of radiation dose on the gel fraction of the hydrogels is shown in Figure 2a.

Even at the lowest applied irradiation dose (5 kGy), relatively high crosslinking efficiency is achieved. With a further increase in the applied doses of 10 and 15 kGy, gel content gradually increases, reaching a maximum value at a dose of 25 kGy. For a radiation dose of 25 kGy, which is generally recommended for the terminal sterilization of medical products, gel content approaches maximum values of 95% for PPG_5_MA compared to 88% for PEG_6_MA, 87% for PPG_6_EG_3_MA, and 78% for PEG_6_PG_3_MA. Upon exposure of the reaction mixture to an absorbed dose of 50 kGy, no significant increase in gel content was observed relative to the value obtained at 25 kGy, indicating that crosslinking reaches saturation at lower doses. Methacrylate-based hydrogels achieve sufficient network formation early, and additional irradiation does not significantly enhance gelation. While high doses can potentially cause degradation through chain scission, our results show that 50 kGy does not induce significant gel degradation. Thus, MA-based hydrogels exhibit limited sensitivity to further irradiation beyond the established crosslinking point. Therefore, hydrogels synthesized at 25 kGy were selected for swelling, DSC, and morphological analysis.

To gain additional insight into the processes that occurred during the hydrogel formation, Fourier Transform Infrared (FTIR) analysis was conducted. FTIR spectra of PG_5_MA, PG_6_EG_3_MA, EG_6_PG_3_MA, and EG_6_MA monomers, as well as corresponding hydrogels and xerogels, are shown in Figure 2b. In all samples of monomers and xerogels, the first broad low-intensity peak around 3400 cm^−1^ is from the bonded OH hydroxyl group. In the single bond region, the absorption bands between 3000 and 2800 cm^−1^ are from the saturated aliphatic C-H bond stretching of CH, CH_2_, and CH_3_ groups. The band at 2970 cm^−1^ is the asymmetric stretching of the CH_3_ group, at 2930 cm^−1^, it is the asymmetric stretching of CH_2_, and the band at 2870 cm^−1^ covers the symmetric stretching of CH_3_ and CH_2_ groups. The existence of two absorption bands for (P)PG_5_MA, one of which, at 2970 cm^−1^ (asymmetric CH_3_ stretching), gradually disappears as the composition changes from (P)PG_6_EG_3_MA and (P)EG_6_PG_3_MA to (P)EG_6_MA, is expected. This is due to the extra methyl group in each PG_5_MA monomer, which differentiates it from the ethylene glycol structures.

In the double bond region, it is evident that the typical carbonyl group (C=O) absorption band appears between 1710 and 1730 cm^−1^. Carbonyl bonds are very polar, and their responses have high intensity, which is located in a unique wavenumber band. Comparing the spectra of the monomers to the xerogels, a shift in the absorption peak of C=O is observed. The monomers contain C=O bonds conjugated to the adjacent C=C bonds (1640 cm^−1^). During polymerization, the alkene functionality becomes alkane, and the C=O bonds are no longer conjugated, resulting in a shift of the absorption peak [45]. The disappearance of the absorption band near 1640 cm^−1^ in the FTIR spectra of all xerogels suggests that the carbon–carbon double bonds (C=C) were consumed, indicating successful chemical crosslinking by gamma irradiation. In contrast, the analysis of hydrogels in the same spectral region is more challenging due to overlapping signals from the bending vibrations of water (HOH), which obscure the C=C region [46]. Besides deformation vibrations (scissor bend) around 1650 cm^−1^, the FTIR spectrum of water also shows an intense and broad band, with a maximum of around 3280 cm^−1^, due to the symmetric–antisymmetric stretching of water molecules. The swollen hydrogels’ FTIR spectra in this region show an increase in overall peak and growth of crest at 3280 cm^−1^ (indicated by the blue arrow).

At room temperature, the equilibrium swelling degrees (Q_e_) of PPG_5_MA, PPG_6_EG_3_MA, PEG_6_PG_3_MA, and PEG_6_MA are 0.1, 2.2, 7.5, and 8.0, respectively (Table 1). FTIR measurements, conducted under the same temperature conditions, show an increase in peak intensity and the growth of the crest at 3280 cm^−1^ in the 3200–3700 cm^−1^ region. This evolution in equilibrium swelling degrees and the intensity of the 3280 cm^−1^ peak are directly related to the hydrophilic nature of the EG units, with more hydrophilic samples exhibiting higher water content.

**Figure 3:**
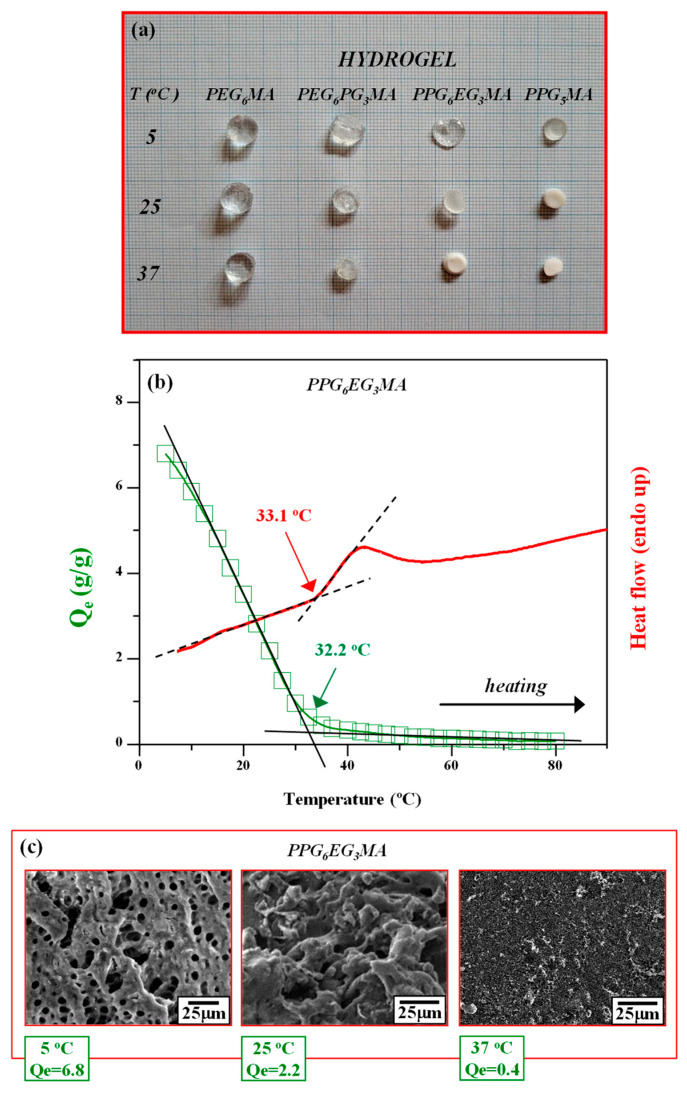
(**a**) Digital photograph of hydrogels swollen to equilibrium in pH 7.4 buffer solution at three different temperatures: 5, 25, and 37 °C. (**b**) Equilibrium swelling degree (Q_e_) and DSC heating scan of PPG_6_EG_3_MA hydrogel in pH 7.4 buffer solution from 3 to 90 °C. (**c**) SEM images of PPG_6_EG_3_MA hydrogel swollen to equilibrium at three different temperatures (5, 25, and 37 °C) in pH 7.4 buffer solution before lyophilization.

A digital photograph of PEG_6_MA, PEG_6_PG_3_MA, PPG_6_EG_3_MA, and PPG_5_MA hydrogels swollen to equilibrium in a pH 7.4 buffer solution at three different temperatures (5, 25, and 37 °C) is presented in Figure 3a. Visual differences in transparency illustrate the thermoresponsive behavior of the hydrogels and align well with the swelling studies shown in Table 1. All hydrogels exhibited significantly higher degrees of swelling at 5 °C, a temperature well below their respective VPTTs. As the temperature increased, a progressive volume decrease was observed, accompanied by increased opalescence, indicating reduced hydration. At 37 °C, PPG_5_MA and PPG_6_EG_3_MA hydrogels, both with VPTTs below this temperature, underwent a pronounced volume contraction, and their appearance shifted to egg-white-like opalescence, which is characteristic of a collapsed polymer network. Notably, the PPG_5_MA hydrogel completely lost its transparency at 25 °C, with no substantial volume change upon further heating to 37 °C. This indicates that the phase transition for PPG_5_MA occurred between 5 and 25 °C, confirming that its VPTT is well below room temperature.

To emphasize the thermoresponsive nature of MA networks with pendant EGPG terminals, the swelling behavior and DSC heating scan (thermogram) of the obtained PPG_6_EG_3_MA hydrogel are shown in Figure 3b. The onset of the endothermic peak in the DSC curve corresponds to the VPTT, i.e., the temperature at which the hydrogel network begins to undergo dehydration and collapse [47,48].

Swelling experiments were conducted under quasi-equilibrium conditions using a very slow heating rate (2.5 °C over 24 h) to ensure sufficient time for the hydrogels to reach equilibrium at each temperature. In contrast, the DSC measurements were performed at a faster heating rate (1 °C/min), which is insufficient to fully reduce kinetic effects. As noted by Manek et al. [49], significantly slower heating rates are recommended to adequately reduce such effects. This discrepancy in heating rates likely explains why the inflection point temperature of the swelling curves (32.2 °C) aligns better with the onset temperature of the thermal transition observed in the DSC measurements (33.1 °C), rather than with the peak or endpoint temperatures of the DSC transition, which are also used as alternative indicators of the VPTT [48,50,51].

The higher hydrophilicity of the EG units compared to that of PG units leads to the shift of VPTT to higher values and a larger swelling capacity of the hydrogels with more EG units within pendant chains. The VPTTs of PPG_5_MA, PPG_6_EG_3_MA, PEG_6_PG_3_MA, and PEG_6_MA, determined from swelling (13.4, 32.2, 47.3, and 70.3 °C) and DSC (13.7, 33.1, 48.3, and 72.1 °C) measurements, follow the same trend as the LCSTs of their monomers (Table 1). A complete set of VPTT results obtained from both methods for all investigated hydrogels is provided in Figures S1 and S2. An increase in VPTT from 32.2 to 47.3 °C, i.e., from 33.1 to 48.3 °C, in the case of MA networks with pendant PG_6_EG_3_ and EG_6_PG_3_ terminals suggests that a customizable and controllable shift in hydrophilicity and VPTT can be obtained by addition and/or variation in the ratio of EG/PG units within the same pendant chain. The ability to create hydrogels with targeted VPTT using structurally uniform pendant chains (instead of structurally distinct ones formed by the copolymerization of two or more monomers) is crucial in pharmaceutical and medical applications due to the simplicity of manufacturing and consistent properties.

Micrographs of the lyophilized PPG_6_EG_3_MA hydrogel discs obtained by SEM are presented in Figure 3c. The SEM images obtained at temperatures far below, a few degrees below, and a few degrees above the VPTT of the sample reveal a strong structure–property relationship. Far below the VPTT, at 5 °C, the equilibrium swelling degree (Q_e_) is 6.8, and the hydrogel exhibits a uniform porous structure with micron-sized pores, enabling high water uptake and swelling. Near the VPTT, at 25 °C (Q_e_ = 2.2), pore distortion and surface defects emerge, indicating reduced porosity. At 37 °C (Q_e_ = 0.4), above the VPTT, the structure collapses into a globular morphology, reflecting dehydration and minimal swelling. These findings suggest that both the initial composition and external factors like temperature play a key role in regulating the swelling behavior and porosity of thermosensitive hydrogels.

## 3. Conclusions

In this paper, methacrylate networks with terminal ethylene glycol–propylene glycol pendant chains were investigated as an alternative to those containing solely ethylene glycol or propylene glycol pendant chains. Compared to pure EG monomers, those containing mixed EGPG units exhibit significantly lower LCSTs, as increasing the PG content reduces overall hydrophilicity and shifts the LCST toward temperatures characteristic of more hydrophobic PG monomers. Herein, we used a mixture of water and ethanol as a bisolvent at various ratios to determine the most suitable settings for successful polymerization. As ethanol content in the binary solvent system increases, the monomer/solvent mixture’s LCST shifts to higher temperatures and solubility increases. This enhances polymerization and crosslinking efficiency during irradiation as the monomer adopts a solvated coil state.

Saturation in gel content, regardless of the hydrogel type, was achieved with an equal volumetric ratio of water and ethanol in the solution. While lowering the ethanol content led to a rapid decrease in gel content, an increase in ethanol content above 50 v% led to a slow decrease in gel content, evident through a significant loss of shape and consistency, making it difficult to handle. In summary, these observations support the conclusion that the synthesis of EG_X_PG_Y_MA- and OPGMA-based hydrogels in a water/ethanol solution with a 50/50 (*v*/*v*) solvent ratio is most likely the best choice. Moreover, the obtained results confirm that the addition of alcohol is necessary in the case of polymer/hydrogel synthesis with thermoresponsive monomers whose LCST is below the temperature at which synthesis takes place. These insights also help define optimal conditions for producing copolymeric hydrogels featuring terminal EGPG pendant chains.

Gel content measurements demonstrate high crosslinking efficiency and successful hydrogel synthesis for the applied dose of 25 kGy when saturation is achieved, and a further increase in absorbed dose results in no significant change in gel content. Since 25 kGy is also the recommended sterilization dose for medical products, it was selected as the most appropriate dose for the synthesis. The findings from the FTIR analysis and gel measurements revealed that polymerization and crosslinking were successfully achieved, while the swelling and DSC revealed a relationship between the monomer composition, the hydrophobic/hydrophilic balance in the monomers, and the VPTT of the resulting hydrogels.

Finally, this study identifies a radiation dose of 25 kGy and a 50/50 (*v*/*v*) water/ethanol solvent ratio as optimal conditions for the radiation-induced hydrogel synthesis of methacrylate networks with EGPG terminal chains.

## 4. Materials and Methods

### 4.1. Materials

The following monomers were used for the radiation synthesis of the homopolymeric hydrogels: (ethylene glycol)_6_ methacrylate (EG_6_MA) (Sigma-Aldrich, Taufkirchen, Germany, M_w_ = 360 g mol^−1^), (propylene glycol)_5_ methacrylate (PG_5_MA) (Cognis UK Ltd.,Monheim, Germany M_w_ = 375 g mol^−1^), (propylene glycol)_6_-(ethylene glycol)_3_ methacrylate (PG_6_EG_3_MA) (Cognis UK Ltd., Monheim, Germany, M_w_ = 566 g mol^−1^), and (ethylene glycol)_6_-(propylene glycol)_3_ methacrylate (EG_6_PG_3_MA) (Cognis UK Ltd., Monheim, Germany, M_w_ = 524 g mol^−1^). According to the specifications given by the producer, the PG_6_EG_3_MA and EG_6_PG_3_MA monomers are methacrylates with a “block” backbone of EG and PG units. Ethylene glycol dimethacrylate (EGDMA) (Sigma-Aldrich, Taufkirchen, Germany, M_w_ = 198.2 g mol^−1^) was used as a crosslinking agent. The chemical structures of the monomers utilized are presented in Table 2. Demineralized water from the Millipore Milli-Q water system (Millipore Corporation, Bedford, MA, USA) was used for all hydrogel synthesis and buffer solution preparation. Absolute ethyl alcohol (C_2_H_5_OH) (Fluka, Buchs, Switzerland, 99.8% purity) was used as a solvent in the hydrogel’s synthesis. A buffer solution at pH 7.4 (with a constant ionic strength of 0.1 mol dm^−3^) was prepared using potassium chloride and potassium mono- and dihydrogen phosphate (Fluka, Buchs, Switzerland). All chemicals were commercial products of the highest available purity and were used as received without further purification. Argon gas (Messer Tehnogas, Serbia, 99.5% purity) was used for degassed reaction mixtures.

### 4.2. Preparation

Hydrogels PEG_6_MA, PEG_6_PG_3_MA, PPG_6_EG_3_MA, and PPG_5_MA were prepared using gamma-radiation-induced polymerization and crosslinking at different radiation doses in various monomer–water/ethanol mixtures. The monomers were dissolved in a water/ethanol mixture by stirring at room temperature for 15 min. The monomer content in the reaction mixture was 10% by weight. To achieve effective crosslinking, EGDMA was added to all reaction mixtures as an efficient crosslinking agent in the amount of 0.5 mol% with respect to the total number of moles of monomers [52]. Four hydrogel libraries (PEG_6_MA, PEG_6_PG_3_MA, PPG_6_EG_3_MA, and PPG_5_MA) were synthesized by varying the ethanol content in the water/ethanol solvent from 0 to 100% in increments of 10%.

Before γ-irradiation, to remove oxygen, the reaction mixtures were saturated with argon gas for 20 min and then sealed in molds of two glass plates separated by a 4 mm thick rubber spacer. Radiation-induced synthesis was performed using a ^60^Co gamma source under ambient conditions, employing the radiation unit for industrial sterilization of the Vinča Institute of Nuclear Sciences, at a dose rate of 0.5 kGy/h to five different absorbed doses (5, 10, 15, 25, and 50 kGy). After irradiation, the samples were annealed at 60 °C for 24 h, then cut into discs (10 mm in diameter, 4 mm thick) and dried at room temperature to a constant weight to obtain xerogels. Each hydrogel formulation was synthesized three times to ensure reproducibility.

### 4.3. UV-Vis Spectroscopy

UV-Vis measurements were performed using a Shimadzu UV-1800 UV-Vis spectrophotometer equipped with a temperature controller (Shimadzu, Kyoto, Japan). The transmittance was recorded at a wavelength of 650 nm in 2 °C increments over a heating run in a temperature range from 5 to 50 °C with a heating rate of 0.2 °C min^−1^. EG_6_MA, EG_6_PG_3_MA, PG_6_EG_3_MA, and PG_5_MA monomers were used to prepare 10 wt% monomer concentrations in various water/ethanol mixtures. The LCST, determined by the cloud point (CP) method, was taken to be at temperatures at which samples exhibit 50% optical transmittance.

### 4.4. Sol-Gel Conversion

To remove any unreacted component, the xerogels obtained after synthesis and drying were subjected to Soxhlet extraction at 40 °C for 48 h. The extracted samples were then further washed in water for one week and, subsequently, dried in a vacuum oven at 40 °C until a constant weight was achieved. The gel fraction was determined gravimetrically using the following mass fraction equation:

Gel Fraction (%) = (W_e_/W_0_) × 100 (1)

where W_0_ represents the initial xerogel weight and W_e_ is the xerogel weight after extraction. The variability of the gel content was assessed using standard deviation.

### 4.5. FTIR Spectroscopy

Infrared spectra were obtained using Fourier Transform Infrared spectroscopy in attenuated total reflectance mode (ATR-FTIR) on a Nicolet IS-50 FTIR spectrometer. Measurements were performed at room temperature over a wavenumber range of 4000–400 cm^−1^ with a resolution of 4 cm^−1^. Spectra were obtained for all monomers (PG_5_MA, PG_6_EG_3_MA, EG_6_PG_3_MA, EG_6_MA), as well as their corresponding xerogels (dried hydrogels), and fully swollen hydrogels equilibrated in water. All FTIR spectra were processed with baseline correction to improve spectral clarity and accuracy.

### 4.6. Swelling Measurement

Swelling measurements were conducted for all hydrogels in a temperature range from 5 to 80 °C in a pH 7.4 buffer solution, which is relevant for biomedical applications. The equilibrium swelling ratio (Q_e_) of the hydrogel was determined gravimetrically and calculated using the following equation:

Q_e_ = (W_e_ − W_0_)/W_0_ (2)

where W_e_ represents the weight of the hydrogel swollen to equilibrium at a predetermined temperature and W_0_ is the weight of the xerogel.

Each xerogel sample was immersed in an excess of pH 7.4 buffer solution and allowed to swell to equilibrium at 5 °C. Afterwards, its weight was measured. Subsequently, the temperature was increased in 2.5 °C increments every 24 h, allowing sufficient time for the sample to reach swelling equilibrium [53], after which weight measurements were taken. The VPTT value was determined from the equilibrium swelling data obtained over the wide temperature range, Q_e_ = f(T), as the temperature where a significant change in slope occurs. The reported VPTT value represents the average obtained from three different specimens of the same hydrogel.

### 4.7. Calorimetric Measurement

Differential Scanning Calorimetry (DSC) was performed using a TA Instruments Q2000 differential scanning calorimeter (TA Instruments, New Castle, DE, USA) over a temperature range of 3–90 °C with a heating and cooling rate of 1 °C min^−1^. All xerogel samples, weighing approximately 5 mg each, were swollen in a pH 7.4 buffer solution and enclosed in aluminum sample holders. A pH 7.4 buffer solution was used as the reference sample. Measurements were carried out under a nitrogen atmosphere. The temperature of the onset of the endothermic peak was taken to be the VPTT value. At this temperature, a disruption of polymer–water interactions occurs, particularly hydrogen bonds, which requires energy input, making it an endothermic process. The absorbed heat corresponds to breaking these hydrogen bonds between polymer chains and water molecules as the polymer transitions from a swollen to a collapsed state [47].

### 4.8. Scanning Electron Microscopy

The morphology of hydrogel specimens was examined using a JEOL JSM-6460 LV scanning electron microscope (SEM) (JEOL, Tokyo, Japan). Sample preparation involved rapid freeze drying in an Edwards Freeze Dryer System (Crawley, UK), which includes a freeze-drying unit and an Edwards E2M8 high-vacuum pump (Crawley, UK). PPG_6_EG_3_MA xerogels were swollen to equilibrium in a pH 7.4 buffer solution at 5, 25, and 37 °C and, subsequently, pre-frozen in a freeze dryer at −80 °C for 24 h under vacuum conditions (~4 mbar). Prior to SEM imaging, the freeze-dried samples were fixed onto graphite stubs and sputter-coated with a 10 nm thick gold layer under low vacuum using a Polaron E5200 SEM (Polaron Equipment Ltd., Watford, UK) auto-coating sputter system.

## Figures and Tables

**Table 1:** LCST of the monomers, equilibrium swelling degrees (Q_e_) of the corresponding hydrogels at three different temperatures (5, 25, and 37 °C), and VPTT obtained through the swelling analysis and and by the DSC (calorimetry) method.

Monomer/ Hydrogel	LCST (°C)	Q_e_ (g/g)			VPTT (°C)	
		5 °C	25 °C	37 °C	Swelling (Inf. Point)	Calorimetry (Onset Point)
(P)PG_5_MA	10.4	2.7 ± 0.4	0.14 ± 0.04	0.1 ± 0.03	13.4	13.7
(P)PG_6_EG_3_MA	14.8	6.8 ± 0.6	2.2 ± 0.3	0.4 ± 0.1	32.2	33.1
(P)EG_6_PG_3_MA	21.1	10.3 ± 0.8	7.5 ± 0.6	4.0 ± 0.4	47.3	48.3
(P)EG_6_MA	-	9.3 ± 0.7	8 ± 0.6	7.1 ± 0.6	70.3	72.1

**Table 2:** Chemical structures, full names, short labels, and molecular weights (M_w_) of the monomers used in the synthesis of the PPG_5_MA, PPG_6_EG_3_MA, PEG_6_PG_3_MA, and PEG_6_MA hydrogels.

Chemical Structure	Full Name	
	Short Label	M_w_ (g/mol)
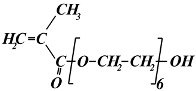	(ethylene glycol)_6_ methacrylate	
	EG_6_MA	360
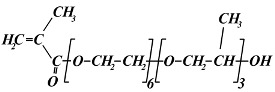	(ethylene glycol)_6_-(propylene glycol)_3_ methacrylate	
	EG_6_PG_3_MA	524
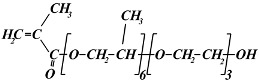	(propylene glycol)_6_-(ethylene glycol)_3_ methacrylate	
	PG_6_EG_3_MA	566
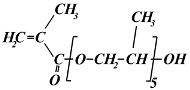	(propylene glycol)_5_ methacrylate	
	PG_5_MA	375

## Data Availability

The original contributions presented in this study are included in the article. Further inquiries can be directed to the corresponding author.

## Supplementary Materials

The following supporting information can be downloaded at: https://www.mdpi.com/article/10.3390/gels11070488/s1, Figure S1. Equilibrium swelling degree (Q_e_) of PPG_5_MA, PPG_6_EG_3_MA, PEG_6_PG_3_MA, and PEG_6_MA hydrogels in pH 7.4 buffer solution as a function of temperature. Figure S2. DSC heating scans obtained for PPG_5_MA, PPG_6_EG_3_MA, PEG_6_PG_3_MA, and PEG_6_MA hydrogels with a heating rate of 1 °C/min.

## Abbreviations

The following abbreviations are used in this manuscript:

EG	Ethylene glycol
PG	Propylene glycol
EGPG	Ethylene glycol propylene glycol
PEG	Poly(ethylene glycol)
PPG	Poly(propylene glycol)
EG_6_MA	Ethylene glycol_6_ methacrylate
EG_6_PG_3_MA	(Ethylene glycol_6_-propylene glycol_3_) methacrylate
PG_6_EG_3_MA	(Propylene glycol_6_-ethylene glycol_3_) methacrylate
PG_5_MA	Propylene glycol_5_ methacrylate
OEGMA	Oligo(ethylene glycol) methacrylate
OPGMA	Oligo(propylene glycol) methacrylate
P(EGPG)MA	Poly(ethylene glycol-propylene glycol) methacrylate
PEG_6_MA	Poly(ethylene glycol)_6_ methacrylate
PPG_5_MA	Poly(propylene glycol)_5_ methacrylate
PEG_6_PG_3_MA	Poly((ethylene glycol)_6_-(propylene glycol)_3_) methacrylate
PPG_6_EG_3_MA	Poly((propylene glycol)_6_-(ethylene glycol)_3_) methacrylate
CP	Cloud point
LCST	Lower critical solution temperature
VPTT	Volume phase transition temperature
Q_e_	Swelling degree
SEM	Scanning electron microscopy
DSC	Differential Scanning Calorimetry
FTIR	Fourier Transform Infrared
HEMA	2-Hydroxyethyl methacrylate
IA	Itaconic acid
EGDMA	Ethylene glycol dimethacrylate
