# Supplementary material for: Radiation-Induced Synthesis of Polymer Networks Based on Thermoresponsive Ethylene Glycol Propylene Glycol Monomers
Source: Gels. 2025 Jun 24;11(7):488. doi: 10.3390/gels11070488 (PMC12294819; doi:10.3390/gels11070488)
Supplement: Supplementary file 1 [file gels-11-00488-s001.zip › gels-3680034-supplementary.pdf]

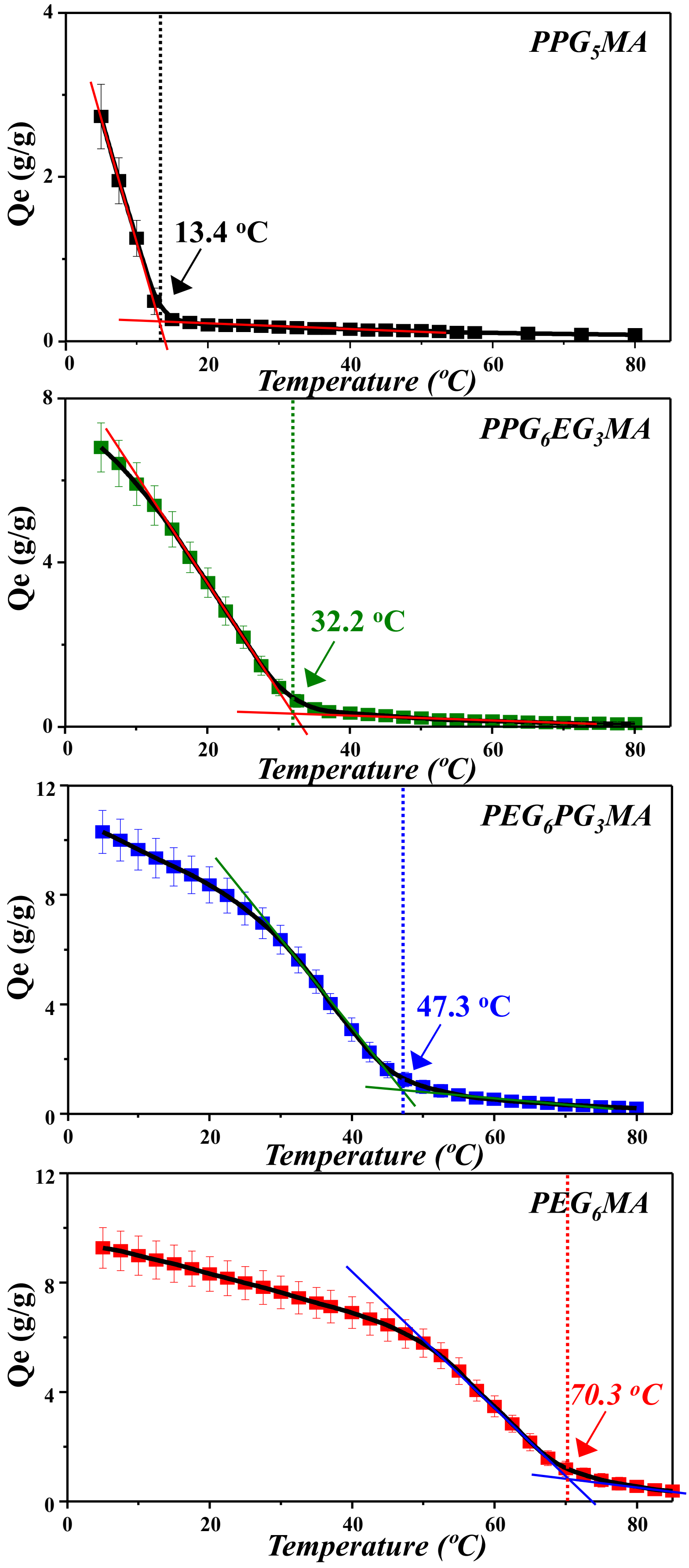

Figure S1. Equilibrium swelling degree (Q_e) of PPG_5MA, PPG_6EG_3MA, PEG_6PG_3MA, and PEG_6MA hydrogels in pH 7.4 buffer solution as a function of temperature.

Figure S1

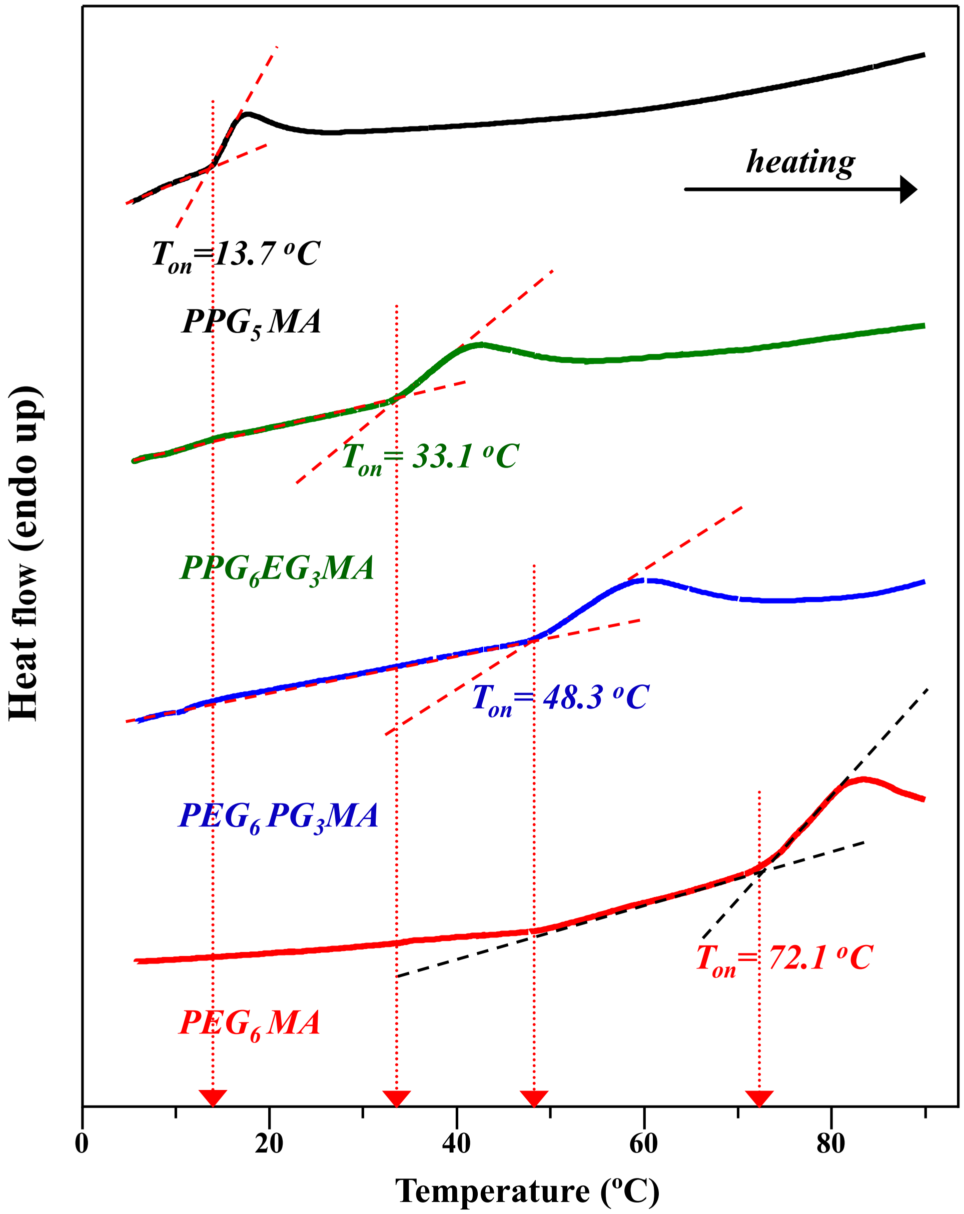

Figure S2. DSC heating scans obtained for PPG_5MA, PPG_6EG_3MA, PEG_6PG_3MA, and PEG_6MA hydrogels with a heating rate of 1 °C/min.

Figure S2
